# Determinants of utilisation differences for cancer medicines in Belgium, Scotland and Sweden

**DOI:** 10.1007/s10198-016-0855-5

**Published:** 2016-12-09

**Authors:** Alessandra Ferrario

**Affiliations:** 1 Department of Social Policy, London School of Economics and Political Science, London, UK; 2 LSE Health, London School of Economics and Political Science, Houghton Street, London, WC2A 2AE UK

**Keywords:** Medicines utilisation, Multilevel mixed-effects data models, Oncology, Managed entry agreements, Pharmaceutical policy, I10, I14

## Abstract

**Background:**

Little comparative evidence is available on utilisation of cancer medicines in different countries and its determinants. The aim of this study was to develop a statistical model to test the correlation between utilisation and possible determinants in selected European countries.

**Methods:**

A sample of 31 medicines for cancer treatment that obtained EU-wide marketing authorisation between 2000 and 2012 was selected. Annual data on medicines’ utilisation covering the in- and out-patient public sectors were obtained from national authorities between 2008 and 2013. Possible determinants of utilisation were extracted from HTA reports and complemented by contacts with key informants. A longitudinal mixed effect model was fitted to test possible determinants of medicines utilisation in Belgium, Scotland and Sweden.

**Results:**

In the all-country model, the number of indications reimbursed positively correlated with increased consumption of medicines [one indication 2.6, 95% CI (1.8–3.6); two indications 2.4, 95% CI (1.4–4.3); three indications 4.9, 95% CI (2.2–10.9); all *P* < 0.01], years since EU-wide marketing authorisation [1.2, 95% CI (1.02–1.4); *p* < 0.05], price per DDD [0.9, 95% CI (0.998–0.999), *P* < 0.01], and Prescrire rating [0.5, 95% CI (0.3–0.9), *P* < 0.05] after adjusting for time and other covariates.

**Conclusions:**

In this study, the most important correlates of increased utilisation in a sample of cancer medicines introduced in the past 15 years were: medicines coverage and time since marketing authorisation. Prices had a negative effect on consumption in Belgium and Sweden. The positive impact of financial MEAs in Scotland suggests that the latter may remove the regressive effect of list prices on consumption.

**Electronic supplementary material:**

The online version of this article (doi:10.1007/s10198-016-0855-5) contains supplementary material, which is available to authorized users.

## Introduction

Managing the introduction of new, high-priced cancer medicines is a challenge for countries at all levels of development. On the one side, payers want to provide access to new and potentially more effective medicines, while on the other they need to ensure the financial sustainability of their health care systems, value for money and an equitable distribution of the available resources.

In Europe, decisions regarding the reimbursement of new high cost medicines are increasingly made using health technology assessment (HTA). While important differences exist in the way individual countries implement HTA, they all include in their analysis and decision-making process information on the efficacy and effectiveness and, to different extents, information on the price of the new medicine. This technique not only enables to determine the cost-effectiveness of a medicine according to the licensed indication, it can also help identifying the patient subgroup in which the medicine is most cost-effective. Limiting access to such a subgroup of patients is one tool countries are using to manage the introduction of new medicines.

Another way to manage entry is to delay the assessment of new medicines. This may be done with an explicit rationing objective, it may be caused by the time involved in conducting HTA but it may also be due to the lack of submission of a pricing and reimbursement dossier by the manufacturer. These factors can lead to a medicine not being reimbursed at all in a particular country, or to reimbursement being limited to a subset of all licensed indications. Lack of a national level positive decision on reimbursement, or lack of a legal requirement to implement such a decision at local level, can lead to disparities in availability of the medicine for patients within countries. In this context, local authorities or hospitals will decide whether or not to fund the medicine, and could possibly lead to no availability at all.

Increasingly, countries are using managed entry agreements (MEAs) to facilitate access while trying to limit budget impact and improve cost-effectiveness in a context of uncertainty [1–3]. The way medicines are financed also has an impact on access, and can influence their uptake. Availability of top-up funding for new high cost medicines can incentivise prescribing and use over medicines that are funded by hospital budgets. The effect of prospective payment systems like diagnostic-related groups is most likely dependent on their design [4]. Special funds earmarked for particular products have been established in some countries. Examples include the cancer drugs fund in England [5], the risk-share scheme for orphan medicines, the rare disease fund in Scotland [6, 7], and the rare disease fund in Belgium [8]. The latter aim to increase availability of high cost medicines that may otherwise be unavailable or whose financing would cause individual institutions financial difficulties.

The setting—ambulatory or hospital—where the medicine is prescribed and dispensed can determine whether a co-payment applies or not, and potentially influence levels of utilisation too. While co-payments for cancer medicines (particularly when these medicines are dispensed in hospitals) are not common in Western European countries, their use and the extent of cost-sharing may influence utilisation. In some countries, third party payers may require prescribing doctors to obtain prior authorisation by a physician designated by the payer, before the medicine can be prescribed.

Beyond pricing and reimbursement, disease burden, demographics, access to timely diagnosis, clinical practice, access to specialist care, whether the disease is a national priority, and cultural factors including defensive medicine also have an impact on use of new high priced medicines and may lead to differential uptake across countries [9–13].

While the factors influencing access and use of cancer medicines have, to a certain extent, been identified and discussed in the literature, less evidence is available on the actual levels of cancer medicine consumption across different countries, and, most importantly, their determinants.

Differences in the use of cancer medicines between selected high income countries have been investigated in an international study on medicine use in 2008/2009 and its update in 2013/2014 [9, 14]. A study on endocrine therapies for breast cancer investigated patterns of use in eight western European countries plus Australia over the period 2001–2012 [15]. A series of comparative longitudinal studies on patient access to cancer medicines in Europe have been conducted covering the time period from 1993 to 2014. These studies looked at differences in expenditure and, for selected medicines, also milligrams or grams per case, and defined daily doses per case [16–18]. Possible determinants of utilisation differences or lack of differences have been investigated using a qualitative approach (benchmarking possible determinants against quantitative data) [9, 15, 19, 20]. A study on utilisation of orphan medicines vs. non-orphan medicines used the *t* test to assess whether an association existed between orphan medicine status and variability in use across countries [21]. Some correlation analysis was conducted in the 2016 update of the study on uptake of oncology medicines in Europe, which noted that uptake of innovative medicines depends largely on the country’s gross domestic product, and the level of health care spending per capita [18]. However, the authors also noted that differences in use across countries with similar economic status exist [18]. Reasons for the limited number of studies analysing consumption of cancer medicines data particularly those applying statistical methods are likely to include, but are not limited to, the difficulties in accessing data on medicines dispensed in hospitals from public sources, and the cost of accessing from private ones. Furthermore, it can be difficult, and sometimes impossible, to assign a numerical value to all possible determinants of use to test as part of a statistical model.

The aim of this study was therefore to test the correlation between utilisation of cancer medicines (mostly dispensed in hospital settings) and possible determinants of utilisation of cancer medicines in selected European countries. The study contributes to the existing literature by providing updated longitudinal evidence on consumption of cancer medicines in Europe, and by developing a longitudinal multilevel model to test the correlation between utilisation and likely determinants adjusting for important covariates.

## Methods

### Sample selection and variable definition

Three countries were selected based on access to data from public sources: Belgium, Scotland and Sweden. Despite the convenience nature of the sample, the three countries represent a suitable study group due to their geographical location (Western Europe), similar gross domestic product and spending on health per capita levels, health system organisation (comprehensive universal health coverage system), and a population size ranging from more than 5 million to 11 million inhabitants in 2013 (Supplementary Data, SD1).

**Fig. 1:**
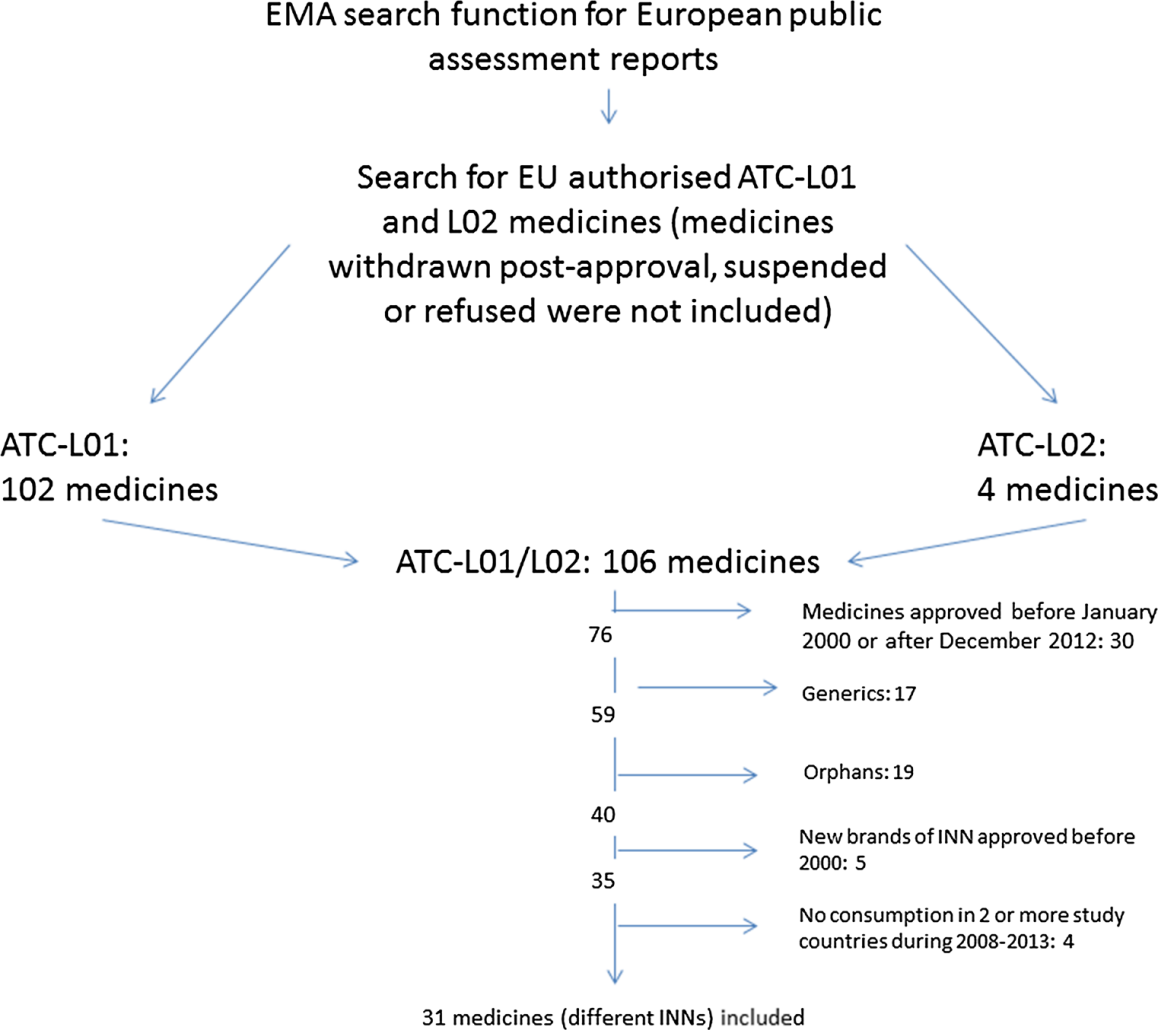
Selection of cancer medicines to be included in the study

Using the anatomic therapeutic chemical (ATC) search function for European Public Assessment Reports available from the website of the European Medicines Agency (EMA), all antineoplastic (ATC-L01) and endocrine (ATC-L02) medicines authorised in the European Union (EU) and the European Economic Area countries (Iceland, Liechtenstein and Norway) were identified. Medicines that were withdrawn post-approval, suspended or refused were not included. The unified list (ATC-L01 and L02) contained 106 medicines (different brand names). I selected all medicines that obtained EU-wide marketing authorisation between 2000 and 2012 (total 76). In an attempt to have a homogenous, yet sufficiently large, sample (at least 30 medicines), I excluded generics (17), orphan medicines (as classified by EMA at the time of data extraction) (19), biosimilars (zero) and medicines approved under exceptional circumstances (zero after excluding orphans) since uptake of these medicines is likely to be influenced by different factors than for the majority of other medicines included. There were five medicines approved with a conditional marketing authorisation in the remaining sample of 40 medicines. I excluded five medicines that were new brands of international non-proprietary names (INNs) approved before 2000. Finally, after extracting data on consumption from the three study countries, I excluded four medicines for which there was no consumption during the study period (2008–2013) in two or more study countries. The final sample of medicines (different INNs) included in the analysis was 31 (Supplementary Data, SD2) (Fig. 1).

Although the subject matter of this study was cancer, I did not include immunostimulant medicines (ATC-L03) because only some of these medicines are indicated for cancer treatment. Further, even if a medicine is indicated for the treatment of cancer, the other indications may not be and the data did not allow to disaggregate medicines use by indication.

### Data sources

Quantitative (e.g. mg of medicines dispensed, date of reimbursement for the first indication) and qualitative (e.g. positive vs. negative reimbursement decision, implementation of MEAs) data were used to build the statistical model. Data on medicines utilisation (pack size, number of packs, strength per quarter or month) in ambulatory and hospital settings by INN and brand (Belgium and Sweden) and by INN only (Scotland) between 2008 and 2013 was obtained from the National Institute for Health and Disability Insurance (INAMI-RIZIV) in Belgium, the Dental and Pharmaceutical Benefits Agency (TLV) and eHälsomyndigheten in Sweden and the National Health Service (NHS) in Scotland. The list of MEAs for each country was sourced from a previous study for Belgium and Sweden [3] and the Scottish Medicines Consortium website for Scotland [22]. Prices per defined daily dose (DDD) were estimated from expenditure data provided by INAMI-RIZIV in Belgium and TLV and eHälsomyndigheten in Sweden, and using historical prices from the British National Formulary in Scotland. DDDs from the Belgian Centre of Pharmacotherapeutic Information [23] were used since the ATC/DDD Index of the WHO Collaborating Centre for Drug Statistics Methodology does not define DDDs for most cancer medicines.

Additional data on the study variables was extracted from the websites of national competent authorities (e.g. HTA reports and ministerial decisions) [24–26], personal contacts with these authorities or clinicians, and from the utilisation data provided by the countries.

The variables extracted included the date when a positive reimbursement decision (Belgium and Sweden) or positive recommendation for use (Scotland) for the first indication of a medicine was made, the number of indications (measured as different types of cancer) covered or recommended for use in each of the study years, use and type of a MEA, setting where the medicine was dispensed and patient co-payments.

While decisions in Belgium and Sweden—relate to reimbursement and in Scotland they relate to use within the national health system, for the sake of simplicity the term used henceforth is ‘reimbursement’ or ‘coverage’. In Sweden, a reimbursement decision is usually made by TLV for outpatient medicines and by the county councils (the latter, in recent years were increasingly made through the NT-council, a body representing all the county councils) for hospital medicines. Some medicines are not assessed by any of these two bodies but recommended as part of national guidelines. National guidelines aimed at supporting resource allocation decisions are developed by the National Board of Health and Welfare (NBHW), these are not clinical guidelines but can and are also used as clinical guidelines. Further, since 2011, national guidelines on breast, prostate, colorectal and lung cancer are developed by professional organisations under the regional cancer centre. Before 2011, each professional group was responsible for cancer care programmes. I therefore checked guidance by the NBHW, the regional cancer centre, and contacted clinicians responsible for the cancer care programmes before 2011.

### Data analysis

The study included a total of nine independent variables. This includes six continuous variables, notably (1) the number of years since a medicine obtained EU-wide marketing authorisation for the first indication; (2) the number of years since a positive reimbursement decision was awarded for the first indication; (3) the median price per DDD; (4) time (year 1–6); (5) total pharmaceutical expenditure per 1000 capita and year (euros); and (6) the average rating of clinical added value across all indications assessed by the independent Drug Bulletin Prescrire (1–7, where 1 represents highest level of added clinical value, 6 the lowest level, and 7 reserved judgment due to insufficient evidence) [27]. Three categorical variables are also included, namely (1) the number of reimbursed indications (measured as different types of cancer, 0–3); (2) the setting where the medicine was dispensed (1 = hospital only, 2 = ambulatory only, 3 = both); and (3) use of a MEA (1 = no MEA, 2 = health outcome based MEA, 3 = financial, 4 = combination), which were modelled as dummies. The all country model included also dummy variables for countries, and interaction terms between countries and time.

I used DDDs per 1000 population to measure utilisation of cancer medicines. In order to compute the total number of DDDs consumed, I calculated the total mg of active ingredient dispensed for a particular INN. I used the DDD defined by the Belgian Centre of Pharmacotherapeutic Information [23] and divided the total mg by the Belgian DDDs.

The resulting longitudinal data set was analysed in Stata 13 using the mixed command to allow for random slopes [28]. To allow for non-linear increase in utilisation over time, I used a non-linear polynomial function with t^2^ as an additional predictor [29]. The between-medicines variability is treated as a random effect (i.e. as a random-intercept term at the medicine level):

$$y_{ijk} = \, \alpha_{jk} + \, \beta_{k} X_{ijk} + \, t \, + \, t^{2} + \, k \, + \, t \times k \, + \, t^{2} \times k \, + \, u_{j} + \, v_{k} + \, e_{ijk}$$

where *i* year, *j* medicine and *k* country, *X*_ijk_ is a vector of all the independent variables included, *u*_j_ is the medicine specific random effect, *v*_k_ is the country specific fixed effect, and *e*_ijk_ is the error term.

Random effects model unobserved between-subject (in this case medicines) variation as random, while fixed effects model unobserved variation between subjects (in this case countries) as constant [28]. Interactions allow for differential increase (slope) between subjects. In this case they allow for time to have a different effect on consumption growth in each country. The logarithm of medicines consumption was modelled because of non-normal distribution of the non-transformed dependent variable.

## Results

### Descriptive analysis

The median time since EU-wide marketing authorisation in the sample was 5.6 years (*N* = 31 medicines, min 1.3 years, max 13.3 years) as of December 2013. At that time, the median time since a positive reimbursement for the first indication was made was 3.9 years (*N* = 30, min 0.45 years, max 10.5 years) in Belgium, 3.7 years (*N* = 16, min 0.23, max 11.2) in Scotland and 4.2 years (*N* = 21, min 0.1, max 12.7) in Sweden.

Some medicines had not been assessed by Scottish Medicines Consortium (1) and neither TLV nor the NT-council nor the NBHW nor professional bodies in Sweden (9); other medicines were assessed and rejected (1 Belgium, 12 Scotland and 1 Sweden) as of December 2013. Until a few years ago, the adoption and utilisation of new cancer medicines in Sweden used to be at the discretion of the oncologists and their institutions. In the last few years, national guidance has increasingly become available through professional bodies, the NBHW, the NT and TLV. Out of the total 31 INNs studied, Belgium recommended at least one indication for 30 of them, Scotland 18 and Sweden 21.

**Fig. 2:**
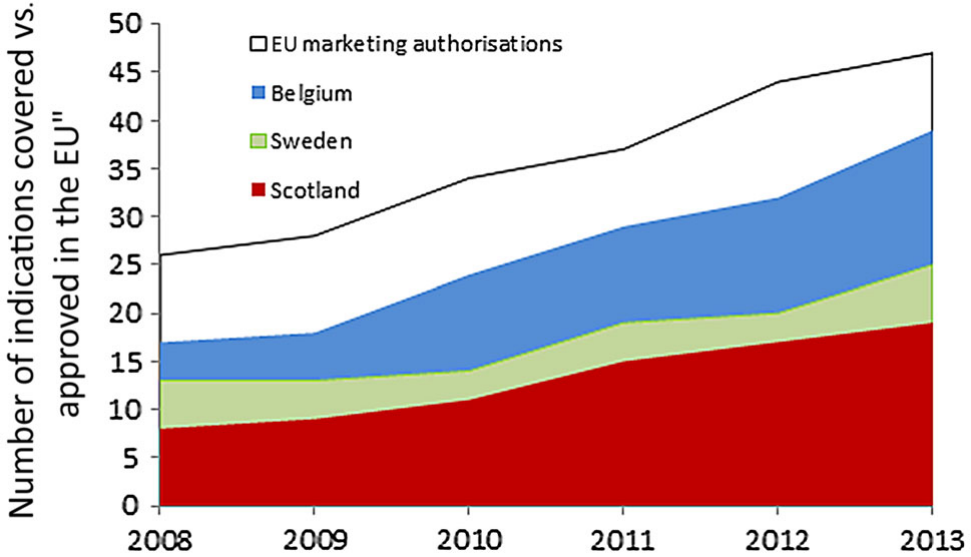
Cumulative number of indications covered vs. approved European Union (EU) indications (both measured as different types of cancer)

Belgium had the highest number of indications covered (*N* = 39, 83% of total indications with EU-wide approval, *N* = 47) as of December 2013, followed by Sweden (*N* = 25, 53% of total) and Scotland (*N* = 19, 40% of total) (Fig. 2). This does not mean that the other indications were available only if the patient paid out-of-pocket, but that there was no national level reimbursement decision. Individual hospitals or local authorities may then decide to make the medicine available to all or selected patients.

In Belgium, cancer medicines are fully reimbursed by compulsory health insurance. Similarly, but not limited to cancer medicines, there are no co-payments or charges on prescription medicines in Scotland. In Sweden, prescription medicines dispensed in hospitals are reimbursed at 100%, while prescription medicines dispensed in pharmacies are subject to a deductible plus a co-payment. Outpatient-orders, when for example the primary care unit has ordered a medicine and dispense it to the patient during the consultation, are not subject to co-payments. Since there were no co-payments in Belgium and Scotland and very minimal co-payments in Sweden (maximum annual co-payment for one year is about EUR 240 (SEK 2200) for all medicines [30]), this variable was not included in the statistical model.

Five, nine, and four medicines were part of a MEA in Belgium, Scotland and Sweden, respectively. These included five combination agreements in Belgium, eight financial and one combination agreements in Scotland and three combination and one health outcome agreements in Sweden.

**Fig. 3:**
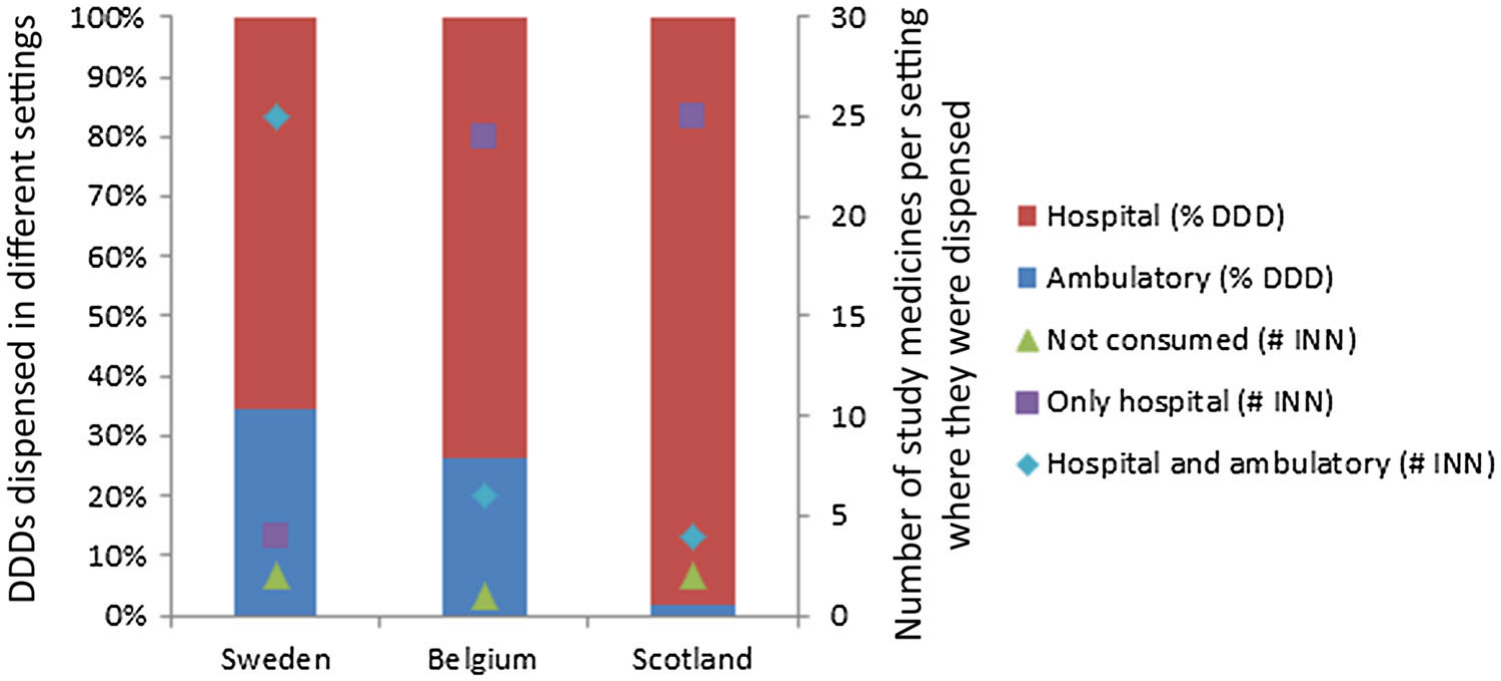
Settings where medicines were dispensed by share of defined daily doses (DDDs) and number of medicines

In all three countries, the largest volume share amongst the medicines in the sample (measured as total number of DDDs) was dispensed in hospital settings (including day care units) in all years. In 2013, this share was highest in Scotland (98%), followed by Belgium (74%) and Sweden (66%). Sweden had the highest number of medicines dispensed in both hospital and ambulatory settings (25), followed by Belgium (6) and Scotland (4) (Fig. 3).

**Fig. 4:**
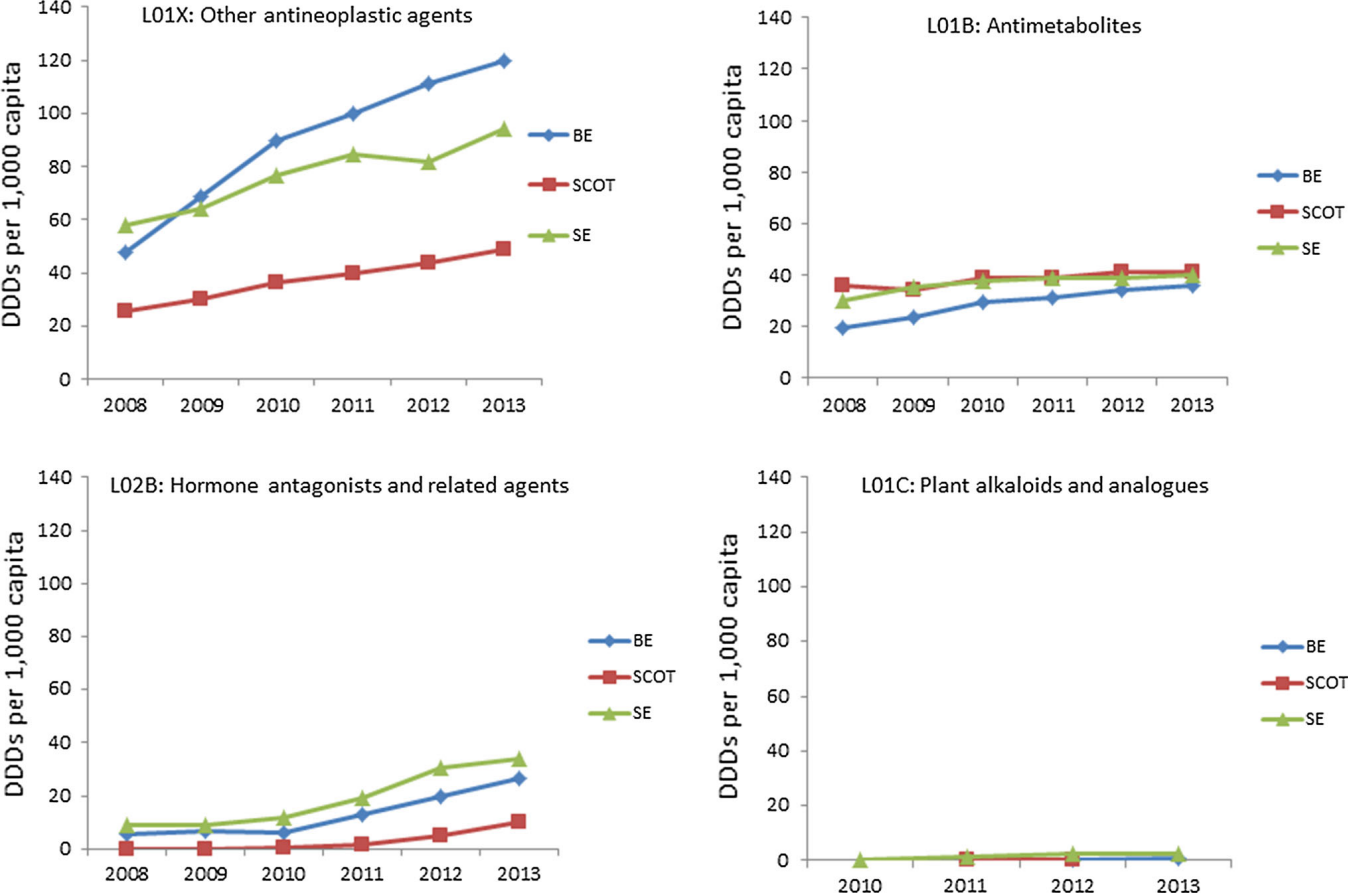
Number of smallest units consumed per 1000 capita

The following figures (Fig. 4) show medicines consumption as the number of DDDs dispensed per 1000 population between 2008 (or the year when utilisation of the medicine was first recorded) and 2013 by ATC-level 3 pharmacological subgroup. Belgium had the highest per capita consumption of ‘other antineoplastic agents’ (L01X), the group to which most study medicines belong (*N* = 23) in all years, while Sweden had the highest per capita consumption of ‘hormone antagonist and related agents’ (L02B) (*N* = 3), and ‘plant alkaloids and analogues’ (L01C), (*N* = 2). Scotland, closely followed by Sweden, had the highest per capita consumption of ‘antimetabolites’ (L01B) included in the study sample (*N* = 3).

The median price per DDD was highest in Belgium (EUR 116.5, min = EUR 2.9, max = EUR 3966.6), followed by Sweden (EUR 90.3, min = EUR 2.4, max = EUR 2724.4), and lowest in Scotland (EUR 88.1, min 3.6, max 5030.2).

The median Prescrire rating across all the 31 medicines included in the study was 5, which corresponds to ‘nothing new’. The medicine with the highest rating was imatinib with a rating of 2, representing ‘a real advantage’, for four of the six indications evaluated. A number of medicines (*N* = 11) had a Prescrire rating of 6, which stands for ‘not acceptable’.

### Statistical analysis: longitudinal mixed-effects model

In the all-country model, the number of indications reimbursed [one indication = 2.6, 95% CI (1.8–3.6); two indications = 2.4, 95% CI (1.4–4.3); three indications = 4.9, 95% CI (2.2–10.9); all *P* < 0.005], and the number of years since marketing authorisation [1.2, 95% CI (1.1–1.4), *P* value <0.05] positively correlated with increased consumption of medicines after controlling for time and other covariates (Table 1). Price per DDD [0.9, 95% CI (0.998–0.999), *P* value <0.05] and the low added clinical value had a regressive effect on consumption [0.5, 95% CI (0.3–0.9), *P* value <0.05]. Having controlled for time and country effects, no correlation was found with the number of years since a positive reimbursement decision for the first indication was given, the existence or not of managed entry agreements, or the level (or the log) of total pharmaceutical expenditure per 1000 capita.

**Table 1:** All-country model

	Exp (β)	(95% CI)		*P* value
Years since EU-wide marketing authorisation	1.202*	1.021	1.405	0.026
Years since positive reimbursement decision	0.998	0.980	1.015	0.795
Number of disease areas covered				
1	2.599**	1.840	3.633	0.000
2	2.425*	1.377	4.259	0.002
3	4.904**	2.192	10.913	0.000
Use of managed entry agreements (baseline: no MEAs)				
Health outcome MEA	0.962	0.393	2.627	0.933
Combination MEA	1.590	0.693	3.653	0.274
Financial MEA	1.539	0.831	2.852	0.171
Setting where the medicine is dispensed (baseline: hospital)				
Ambulatory	0.460	0.156	1.368	0.163
Hospital and ambulatory	0.720	0.495	0.954	0.087
Price per DDD	0.999*	0.998	0.999	0.001
Prescrire rating	0.545*	0.337	0.881	0.013
Pharmaceutical expenditure per 1000 capita	1.000	1.000	1.000	0.837
Year	0.925	0.605	1.412	0.716
Year^2^	1.019	0.945	1.098	0.626
Country (baseline: Scotland)				
Sweden	4.711*	0.343	57.111	0.034
Belgium	4.393	0.372	3.785	0.255
Sweden × year	1.185	0.593	1.896	0.772
Belgium × year	1.062	0.790	1.188	0.839
Sweden × year^2^	0.969	0.868	1.091	0.761
Belgium × year^2^	0.973	0.003	5469.815	0.642
Constant	3.896	1.000	1.000	0.712

* EU* European Union,* MEA* managed entry agreements,* DDD* defined daily dose,* CI* confidence interval

* *P* < 0.05, ** *P* < 0.001

Results for the country level models showed that the following variables were positively correlated with increased consumption: the number of disease areas covered in all countries, the number of years since EU-wide marketing authorization in Scotland and Sweden, the use of managed entry agreements in Scotland, and the setting where the medicine was dispensed in Sweden. The variables that negatively influenced consumption were: the price per DDD in Belgium and Sweden and the low value of a medicine in Scotland (Table 2).

**Table 2:** Individual country models

Belgium	Exp (β)	(95% CI)		*P* value
Years since EU-wide marketing authorisation	0.890	0.653	1.213	0.461
Years since positive reimbursement decision	1.284	0.900	1.833	0.168
Number of disease areas covered	1.000	1.000	1.000	
1	2.648**	1.788	3.923	0.000
2	3.876**	2.119	7.090	0.000
3	6.802**	3.095	14.951	0.000
Use of managed entry agreements (baseline: no MEAs)				
Combination MEA	1.043	0.389	2.797	0.934
Setting where the medicine is dispensed				
Hospital and ambulatory	0.595	0.112	3.146	0.541
Price per DDD	0.998**	0.997	0.999	0.000
Prescrire rating	0.610	0.366	1.017	0.058
Pharmaceutical expenditure per 1000 capita	1.000	1.000	1.000	0.480
Year	1.049	0.744	1.481	0.784
Year^2^	0.972	0.916	1.033	0.363
Constant	1882.717	0.001	3,399,451,731.077	0.305

Sweden	Exp (β)	(95% CI)		*P* value
Years since EU-wide marketing authorisation	1.361**	1.139	1.616	0.000
Years since positive reimbursement decision	0.902	0.787	1.034	0.140
Number of disease areas covered				
1	2.316*	1.279	4.221	0.006
2	3.320	1.000	11.023	0.050
Use of managed entry agreements (baseline: no MEAs)				
Health outcome MEA	3.287	0.826	13.197	0.091
Combination MEA	0.888	0.326	2.411	0.816
Setting where the medicine is dispensed				
Ambulatory	0.519	0.174	1.537	0.238
Hospital and ambulatory	1.685*	1.048	2.713	0.031
Price per DDD	0.998**	0.954	2.691	0.000
Prescrire rating	0.784	0.522	1.174	0.242
Pharmaceutical expenditure per 1000 capita	1.000	1.000	1.000	0.103
Year	4.688	0.779	28.078	0.090
Year^2^	0.788	0.595	1.045	0.090
Constant	77652.576	0.044	140,363,314,266.971	0.125

Scotland	Exp (β)	(95% CI)		*P* value
Years since EU-wide marketing authorisation	1.289*	1.115	1.490	0.001
Years since positive reimbursement decision	1.011	1.000	1.022	0.061
Number of disease areas covered				
1	1.832	0.967	3.471	0.063
2	1.180	0.322	4.329	0.803
3	38.205**	2.395	609.506	0.010
Use of managed entry agreements (baseline: no MEAs)				
Combination MEA	1.100	0.272	4.446	0.894
Financial MEA	3.249*	1.554	6.791	0.002
Setting where the medicine is dispensed				
Hospital and ambulatory	1.174	0.602	2.288	0.638
Price per DDD	0.998	0.995	1.001	0.193
Prescrire rating	0.552*	0.367	0.832	0.004
Pharmaceutical expenditure per 1000 capita	1.000	1.000	1.000	0.710
Year	1.066	0.788	1.440	0.679
Year^2^	0.976	0.935	1.019	0.267
Constant	2.598	0.028	240.853	0.679

* *P* < 0.05, ** *P* < 0.001

## Discussion

Overall, Belgium and Sweden had the highest level of consumption (measured as DDD/1000 capita) for non-orphan cancer medicines that obtained EU-wide marketing authorisation between 2000 and 2012. Belgium had the highest absolute number of DDDs consumed per 1000 capita in 2012 and 2013. This does not seem to be explained by the burden of disease, since Sweden has generally a lower incidence rate than Belgium and Scotland for a number of cancer (apart for melanoma), and Scotland has the highest incidence among the three countries for a number of cancers (e.g. breast, bronchus and lung, liver, oesophagus, pancreas and stomach) (Supplementary Data, SD3). In Sweden and Scotland, medicines used in hospitals are financed through the hospital budget, and may be used before a national level decision on reimbursement is made. In contrast, in Belgium, with the exception of compassionate use, pricing and reimbursement had to be completed before doctors could prescribe a medicine in hospital settings as of December 2013. However, once this is completed, hospital medicines are reimbursed separately by INAMI and are not part of the fixed hospital budget. This may explain why Belgium has higher per capita consumption than Sweden between 2011 and 2013, and generally a higher consumption than Scotland despite the latter often having a higher disease burden.

The 2010 report to the United Kingdom (UK) Department of Health used rankings to compare use of selected medicines across various therapeutic areas among 14 OECD countries in 2008/2009 [9]. An update of the 2010 report was released in 2014 providing data for 2012/2013 [14]. The two analyses included the UK and Sweden but not Belgium. Although one cannot really compare the results of this study to the UK study due to differences in the medicines included, countries studied, and methods of analysis, one can at least observe that utilisation of cancer medicines in Sweden was usually higher than in Scotland in this study. In the UK Department of Health study, Sweden ranked 9th in terms of cancer medicines consumption and the UK ranked 10th (with some differences within the cancer class, e.g. use of endocrine therapies was higher in the UK than Sweden). Further, this study confirms the importance of HTA outcomes, included in the model as years since a positive reimbursement decision was made and indications covered, in determining levels of utilisation. This study also confirms the absence of correlation between, in our case pharmaceutical expenditure per capita, in the case of the report health expenditure per capita, and utilisation.

The number of indications covered positively correlated with increased consumption in all models although not all levels of increase were significant. The effect was smallest in Sweden possibly because before 2010, and, to a certain extent still at the end of the study period (2014), decisions on whether to fund or not a cancer medicine have been made by the respective county councils in the absence of national level guidance. Today there is increasing coordination in decision-making thanks to the centralisation of cancer in six centres of excellence, and closer collaboration between TLV and NT- council. The likely impact of coverage decisions on prescribing and consumption was also mentioned in a longitudinal study on endocrine therapies [31], and the role of reimbursement and funding arrangements for governing access to myeloma treatment in England has been highlighted [32].

Time since EU-wide marketing authorisation had a positive effect in all countries but Belgium. One explanation could be that, in Belgium, reimbursement is a more important factor than years since marketing authorisation. Use in both hospital and ambulatory settings vs. hospital only had a positive effect in Sweden, where it applies to most medicines included in this study. Interestingly, the price per DDD had a regressive effect in Belgium and Sweden, but not in Scotland. One possible explanation for the lack of negative impact of prices in Scotland may be the implementation of MEAs (all with a financial component for the medicines with MEA in this sample), which off-set the high list price per DDD. Low value medicines had a regressive effect on consumption in Scotland. The 2016 update of the study on the uptake of new cancer medicines in Europe observed a correlation between the European Society for Medical Oncology Magnitude of Clinical Benefit Scale (ESMO-MCBS) and actual uptake; however, this was not statistically significant, and the number of medicines represented for most non-curative score levels was very small (score 1, *n* = 0; score 2, *n* = 3; score 3; *n* = 3, score 5, *n* = 1), only score 4 had 10 medicines represented [18].

Finally, it is worth noting that large differences in access between and within country are not limited just to medicines but, as shown by an analysis by the OECD, they affect a number of medical procedures [33].

Not all possible determinants of utilisation of cancer medicines are readily convertible into a numeric value that can be tested as part of a statistical model. Examples include cultural factors and clinical practice. Other determinants may be measurable but not readily available at medicine level between 2008 and 2013. For example, inclusion of access to timely diagnosis could be measured by looking at the average stage at which patients are diagnosed. However, the required data for different countries is not simple to obtain, and several medicine-indication combinations and would require utilisation data by indication. This study therefore had to limit the number of variables included to those for which data was available at the required level and frequency in the three study countries.

There are a number of limitations in our study. First, it was not possible to include incidence of different types of cancer in the model. The majority of the medicines included in the sample were approved for the treatment of different cancer indications that are associated with different incidence levels. Only availability of utilisation data by ICD-10 code would have therefore enabled to link use with incidence in a reliable way. Second, it is well-known that list prices (e.g. British National Formulary) and undiscounted expenditure figures do not reflect what health payers pay for medicines [34]. Nevertheless, list prices are still the starting point for negotiation, and the presence of special pricing arrangements at national level is captured by the MEA variable. Further, it would have simply been impossible to access real discounted prices on 31 cancer medicines in three different countries. Third, again due to lack of data at indication level but also lack of cost-utility estimates for all the medicines in each country, I could not include in the model the incremental cost-effectiveness ratio estimated for different indications in each country. Fourth, the number of dispensed doses does not necessarily mean that they were all consumed. Considering the high cost of new cancer medicines and the severity of cancer, it is unlikely that wastage will have significantly affected the results. Fifth, I did not have access to dispensing data at sub-national, and, therefore, could not control for differences in dispensing at that level, which may be significant.

## Conclusions

Access to new medicines ought to be targeted to medicines that bring meaningful added value to patients in comparison to existing therapies. Use of medicines with modest therapeutic improvement, mostly at higher prices than existing treatments, draws resources away from potentially more effective interventions. It is therefore important that competent authorities assess added therapeutic value and enable access to medicines with high value and limit access to those with low value. This study showed that the most important correlates of increased medicines utilisation in a sample of cancer medicines introduced in the past 15 years, were medicines coverage and time since EU marketing authorisation. Prices had a negative effect on consumption, meaning that they can represent a barrier for access. The lack of a regressive effect of prices on consumption in Scotland, and the positive impact of financial MEAs, suggests that the latter may remove the regressive effect of list prices on consumption. Scotland was also the only country where low clinical added value of a medicine was correlated with reduced consumption, suggesting that existing entry arrangements in place, particularly HTA and clinical guidelines, seem to guide towards use of high value products and limiting access to low value ones. However, it is also important to note that Scotland had the lowest level of consumption for most medicines, raising the question as to whether the other two countries have too generous access requirements or whether the former’s are too restrictive. An analysis of patient level data including diagnosis and  prescribing information could help answering this question.

## Electronic supplementary material

Below is the link to the electronic supplementary material.

Supplementary material 1 (XLSX 74 kb)
